# Variety of *Clostridioides difficile* Ribotypes in CDI Patients in Las Vegas, NV

**DOI:** 10.3390/germs16010002

**Published:** 2025-12-23

**Authors:** Amber Consul, Mohamad Mubder, Samrawit Misiker, Shadaba Asad, Kimberly D. Leuthner, Chia-Dan Kang, Yassin Shams Eldien Naga, Chad L. Cross, Ernesto Abel-Santos

**Affiliations:** 1 Department of Chemistry and Biochemistry, University of Nevada–Las Vegas, Las Vegas, NV 89154, USA; 2 University Medical Center of Southern Nevada, Las Vegas, NV 89102, USA; 3 Department of Epidemiology & Biostatistics, School of Public Health, University of Nevada–Las Vegas, Las Vegas, NV 89154, USA; 4 Nevada Institute of Personalized Medicine, University of Nevada–Las Vegas, Las Vegas, NV 89154, USA

**Keywords:** C. *difficile*, CDI, ribotyping, Las Vegas

## Abstract

**Objective:**

Although Las Vegas is a major tourist hub, it is not among the counties that are under CDC surveillance for *Clostridioides difficile* infection (CDI), a major nosocomial infection. To determine the distribution of *C. difficile* ribotypes in the Las Vegas area, we collected stool samples from CDI-positive patients at the University Medical Center (UMC).

**Methods:**

We included adult patients diagnosed with CDI and provided informed consent. *C. difficile* was isolated from the stool samples and ribotyped. Demographic information was also obtained and analyzed. All information was compared to the surveillance data from the CDC.

**Results:**

We identified more frequently in male patients than in the CDC data. Less than half of the patients used antibiotics prior to the infection. We observed several comorbidities in our patient sample pool, with cardiovascular disease and diabetes being the most prevalent comorbidities. Hypervirulent *C. difficile* strain 027 was the most prevalent ribotype. Except for two samples of ribotype 076, all other samples represented unique singlet ribotypes. Four of these ribotypes (160, 302, 363, and 813) have not been explicitly reported in humans.

**Conclusions:**

Due to the unique environment created by the tourism industry in Las Vegas, this population is exposed to national and international visitors. This study shows the pre-COVID landscape of *C. difficile* ribotypes in Las Vegas and offers valuable insights into the varieties of *C. difficile* that are currently infecting this community.

## 1. Introduction

*Clostridioides difficile* is the primary cause of antibiotic-associated diarrhea [1–3]. In the US alone, *Clostridioides difficile* infection (CDI) causes approximately 20,000 fatalities each year, with associated healthcare costs of approximately USD 6.3 billion. Outbreaks caused by hypervirulent *C. difficile* ribotypes are associated with greater mortality rates [4,5]. An additional concern with *C. difficile* is that some ribotypes can cause zoonotic infection [6]. Indeed, *C. difficile* strains associated with human CDI have been detected in both production and companion animal samples [7–9].

CDI is a unique bacterial infection that is frequently triggered by antibiotics [10]. Most *C. difficile* strains are resistant to multiple antibiotics, including clindamycin, β-lactams, and fluoroquinolones [11,12]. The incidence of CDI has increased with the emergence of PCR ribotypes 027, 017, and 078. These lineages are more transmissible and can cause more severe infections [13].

CDC-accepted guidelines define healthcare-acquired CDI (HA-CDI) as infections occurring >48 h after hospital admission or within 4 weeks of discharge from a healthcare facility [14]. On the other hand, community-acquired CDI (CA-CDI) is defined when symptoms occur within 48 h of hospital admission or following a period of at least 12 weeks with no hospitalization. The United States does not have a national *C. difficile* ribotypetracking system. Instead, the CDC monitors only 35 counties across ten states [15]. An unrelated Texas study tracked *C. difficile* ribotypes over the course of seven years. Over the course of the study, a decline was observed in the prevalence of ribotypes 027, 001, and 078-126, with concomitant increases in ribotypes 106 and 054 and the appearance of novel ribotype 255 [16].

A long-term study conducted at the Tufts Medical Center noted a consistent decline in the occurrence of ribotype 027. Despite this decrease, ribotype 027 remained the most frequent isolate, followed by ribotypes 014–020 and 106 [17]. Another study out of Arizona demonstrated similar trends, with ribotypes 027 and 106 being the most prevalent [18].

Travel destinations that attract global visitors can facilitate the rapid spread of infectious diseases [19,20]. However, neither Las Vegas nor any of its neighboring counties are among the counties under CDC surveillance. The lack in epidemiological CDI data in Las Vegas creates an international vulnerability since thousands of tourists visit annually. This transient population can both bring new *C. difficile* strains to the city and, in turn, distribute these strains across the world. In this study, we aim to detect the prevalence of *C. difficile* ribotypes and provide a snapshot of the pre-COVID CDI patient population in Las Vegas.

## 2. Materials and Methods

### 2.1. IRB Protocols and Stool Collection

Stool sample collection was approved by the University Medical Center (UMC) Institutional Review Board (UMC-2019-193). Inclusion criteria for this study were: (1) adult patients aged 18 years or older, (2) patients diagnosed with C. difficile infection via stool PCR test on admission or during hospitalization, and (3) patients or their direct relatives who were able and willing to provide informed consent. Exclusion criteria were as follows: (1) patients under the age of 18 years, (2) patients unable to provide informed consent, and (3) incarcerated patients. Written informed consent was obtained to allow the examination of stool samples already acquired for diagnosis. Demographic information, hospital admissions, antibiotic use, CDI severity, and comorbidities were obtained for all the patients.

The biological samples were de-identified at the UMC and sent to the Abel-Santos laboratory. Access to these de-identified demographic data was granted to the co-investigators for analysis. The data and results were stored on a UMC computer system.

### 2.2. Isolation and Typification of C. difficile from Stool Samples

All growth media and supplements for C. difficile cultivation were obtained from Becton Dickinson (Sparks, MD, USA).

Stool samples were stored at 4 °C and tested within 24–48 h of arrival at the Abel- Santos laboratory. Stool aliquots (~0.25 g) were homogenized in 5 mL of autoclaved water and heated to 68 °C for 30 min prior to plating on CDSA agar. The plates were incubated under anaerobic conditions (5% CO_2_, 10% H_2_, and 85% N_2_) for 48 h. Three colonies that grew on CDSA were picked at random and screened by PCR for *tcdA* and *tcdB* [21,22]. Positive colonies were grown anaerobically in BHI broth for 24 h. Glycerol stocks were prepared and stored at −80 °C.

### 2.3. Molecular Detection of C. difficile, Toxins, and Ribotyping

Primers were obtained from Integrated DNA Technologies (IDT, Newark, NJ, USA). EconoTaq Plus 2X Master Mix from Biosearch Technologies (Alexandria, MN, USA) was used for all nonfluorescent PCR amplifications. PCR Master Mix for fluorescein amidite (FAM)-tagged primer set and Phusion PCR Master Mix with HF buffer were obtained from New England Biolabs (Ipswich, MA, USA). The PCR samples were purified using a Qiagen PCR Purification Kit (Hilden, Germany). Aliquots of bacterial samples putatively identified as *C. difficile* were used as PCR targets to amplify the 16s and 23s rRNA genes. The amplicons were sent to the Nevada Genomic Center for capillary gel electrophoresis. The generated FASTA files were subsequently sent to the *Clostridioides difficile* Ribotyping Network for England and Northern Ireland (CDRN) for ribotype identification.

### 2.4. Statistical Analysis

A chi-square test of homogeneity was used to compare the data from the Las Vegas Valley found in this study (UMC dataset) with the national surveillance data (CDC dataset). Homogeneity of proportion tests were also carried out to separately compare the UMC dataset to the CDC-CA and CDC-HA datasets. An overall test of proportions was first performed, followed by post hoc analyses. All *p*-values for these comparisons were calculated using a robust Monte Carlo resampling procedure with 10,000 replicates.

## 3. Results

### 3.1. Demographic Analysis

In our study cohort (n = 25), the patients’ ages ranged from 22 to 79 years, with an average age of 48 years (Table 1). No significant differences in patient age were found between our sample sets and reported CDC data.

Most patients were from the Las Vegas and the surrounding areas. Three patients had recurrent CDI, while the rest had primary infections. Interestingly, only half of the HA-CDI cases had received antibiotics in the preceding 12 weeks, while less than 10% of the CA-CDI cases had received similar treatments. Comorbidities were prevalent in the cohort, with 22 patients having diagnosed comorbidities. However, these findings were not statistically different from the comorbidity data reported by the CDC. Although not significantly different from CDC-reported comorbidity rates, our patient pool demonstrated higher rates of cardiovascular disease and diabetes. CDI severity varied within our cohort, with 16 cases classified as mild, five as moderate, and two as severe.

In the UMC patient group, there was a notable disparity in CDI cases between sexes, with males comprising 80% of CA-CDI cases and 64% of HA-CDI cases. This significantly contrasts with published CDC data, where males make up a smaller proportion [15].

### 3.2. C. difficile Ribotypes Observed

**Figure 1.**
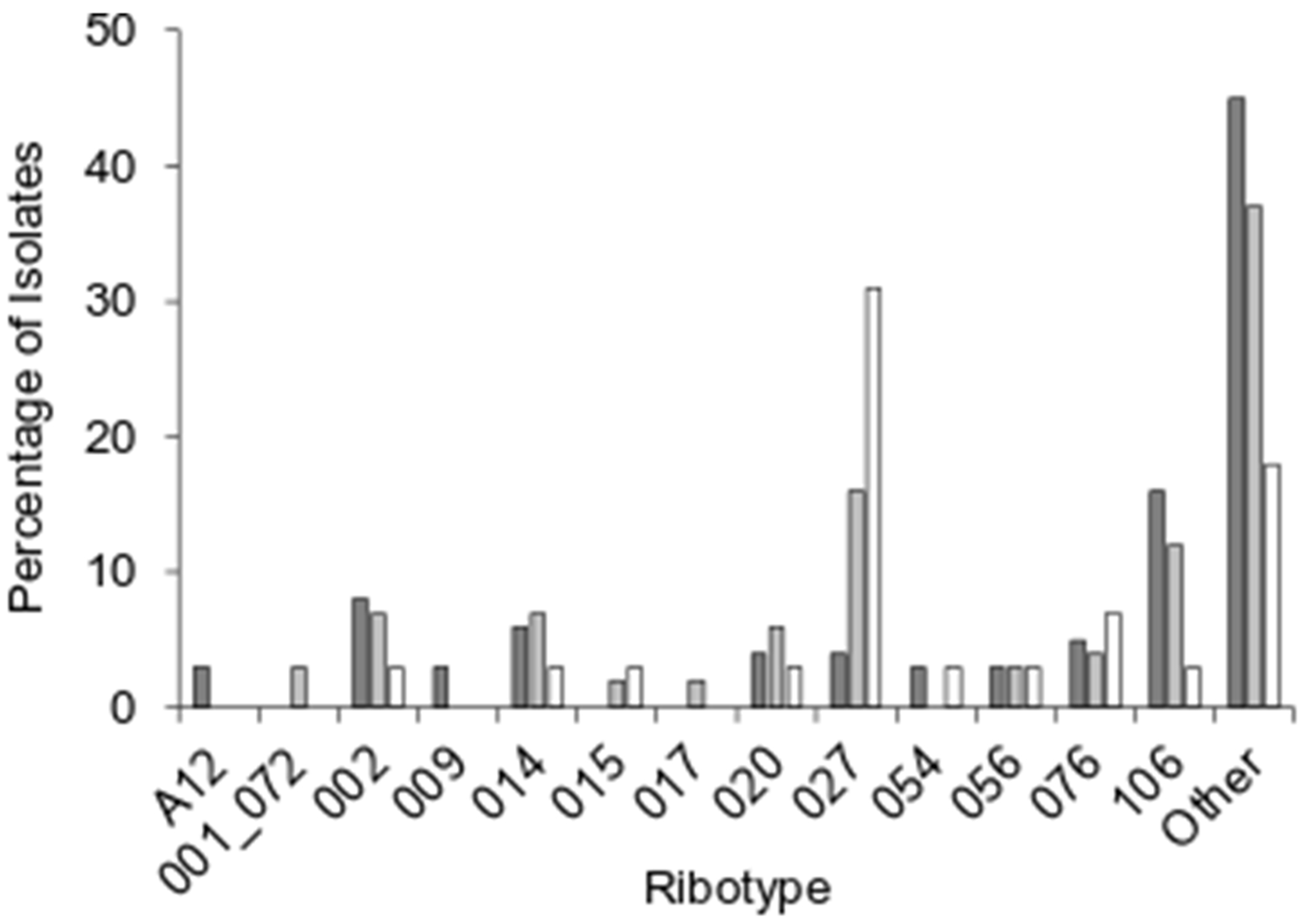
Percentage of ribotypes reported in the community-acquired CDC data set (dark grey columns), healthcare-acquired CDC data set (light grey columns), and UMC data set (white columns). The disparities between ribotypes 027 and 106 in the different patient populations can be observed. CDC data from 2018 was used, for comparison as the ribotype reporting was discontinued the following year [15].

Bacterial colonies picked at random from the CDSA plates were confirmed to be *C. difficile* by 16S and 23S rRNA sequencing. All putative *C. difficile* colonies also encoded for both TcdA and TcdB. Ribotype 027, a hypervirulent strain, was the most frequently identified ribotype in the patient group (Table 2). Both the HA-CDI and CA-CDI patient groups within the UMC population had a significantly higher incidence of ribotype 027 than the CDC data (Figure 1). In contrast, ribotype 106 appeared to be under-represented in the UMC patient population. Except for two samples of ribotype 076, all other samples represented unique singlet ribotypes. Four of these ribotypes (160, 302, 363, and 813) have not been explicitly reported in humans.

## 4. Discussion

The absence of a statistically significant age difference between our study population and CDC data suggests that the age distribution of CDI cases in our region aligns with the national trends. Owing to the small sample size of the UMC dataset, four statistical analysis categories (cardiovascular, cardiopulmonary, malignancy, and transplant) were aggregated. Compared to the CDC-reported figures, the distribution of comorbidities in our patient population did not show a statistically significant difference (*p* = 0.0883), despite the apparently higher rates in the cardiovascular disease and diabetes categories.

Our data highlight the differences in antibiotic use between the HA and CA cases. Due to differences in information reporting, our data could not be compared to CDC’s findings, as their report indicated the number of patients who had received antibiotics but did not differentiate between patients who had not received prior antibiotic therapy and those who did not.

Potential gender-related variations in CDI prevalence within our region may be influenced by behavioral or genetic factors. Our results contrast with the trends reported in the literature, which show a higher incidence of CDI in women than in men when CDI is the primary diagnosis [23–26]. It is important to note that individual risk factors, antibiotic use, and other variables can influence the likelihood of *C. difficile* infection in both men and women. One study postulated that the higher incidence of CA-CDI observed in women could be due to higher outpatient antibiotic prescription rates in women than in men; however, this was not observed in our CA-CDI cases [26]. Other studies have indicated that the epidemiology of CDI, including sex distribution, can vary significantly based on geographic location, age group, and healthcare settings [27,28]. In some analyses, male patients were found to have a longer median hospital length of stay and higher mortality rates than female patients with CDI [27].

Strains from the most common *C. difficile* ribotype found (027) are considered to be hypervirulent and cause severe infections. Strains from the second most prevalent ribotype (076) is not considered to be as virulent as ribotype 027 but are still linked to poor outcomes. All other ribotypes found were singlets with varying degrees of virulence.

*C. difficile* ribotype 027 was significantly more abundant in the CDI population. However, the non-027 ribotypes were remarkably diverse, some of which were novel to the United States. Ribotypes 027, 014, 078, 002, and 126 identified in our patient sample set are notable for their zoonotic potential since they have been identified in both humans and livestock [29]. Ribotypes 137, 160, and 220 have been reported in other countries, whereas ribotypes 302, 363, and 813 have not been described in the literature, indicating limited worldwide information on these ribotypes (Table 2).

The high prevalence of *C. difficile* ribotype 027 in both CA-CDI and HA-CDI patients could have various causes and significant implications. Inappropriate antibiotic usage can disrupt normal gut flora, providing a niche for the proliferation of *C. difficile*. This is further aggravated because ribotype 027 might have increased sporulation rates compared to other strains [30]. *C. difficile* spores are resilient and can survive for long periods on surfaces. Inadequate environmental cleaning and disinfection, particularly in healthcare settings, may contribute to the persistence and spread of this ribotype.

Ribotype 027 has frequently been associated with healthcare settings, where it can spread more easily due to the high density of susceptible individuals, including those with prolonged hospital stays, immunocompromised patients, and those undergoing antibiotic treatment. Hence, regions with larger healthcare facilities may have a higher prevalence of this ribotype because of the higher antibiotic prescription and hospitalization rates.

## 5. Conclusions

Our study provides valuable insights into the epidemiology of CDI in the study area. In our examination of CDI in the Las Vegas patient cohort, we found a significant prevalence of comorbidities. The presence of these health issues not only complicates the clinical management of CDI but also underscores the importance of understanding the prevalence of *C. difficile* strains.

The prevalence of hypervirulent ribotype 027 in the Las Vegas region emphasizes the importance of targeted intervention against this hypervirulent ribotype. The underrepresentation of ribotype 106 in our patient population raises questions regarding regional differences or random variations.

A significant number of CDI cases in our study involved patients with no recent antibiotic exposure, highlighting the possibility of other transmission pathways or environmental sources of *C. difficile*. For example, proton pump inhibitors (PPIs), which reduce stomach acidity and affect gut bacterial control, allow *C. difficile* spores to survive and multiply in the gut [31]. However, delimiting these risks is challenging. Indeed, in our patient sample, only three individuals were on PPIs before their CDI diagnosis, and all were treated with antibiotics within three months of their diagnosis.

Our results run contrary to CDC data that shows women to be more susceptible to CDI than men. These differences might be due to our smaller patient population or to the unique features of Las Vegas as an adult tourist destination. Further research is essential to elucidate the reasons for gender-based differences in CDI incidence.

Owing to the unique environment created by the tourism industry in Las Vegas, this population is exposed to national and international visitors. Effective monitoring of transient areas can contribute to identifying public health concerns such as CDI on a global scale, underscoring the significance of interconnectedness in disease transmission. Because international travel networks can influence disease dynamics, monitoring such destinations aids in recognizing threats and formulating strategies to mitigate potential outbreaks. The clinical benefits of a granular study such as this one will help identify the prevalence of epidemic strains present in the Las Vegas community.

## Figures and Tables

**Table 1.** Comparison of 2019 CDC ribotyping data to UMC patient study.

	CDC Data (%)	UMC Data (%)	Relevant Statistical Analysis
Community Acquired			
Total community acquired cases	7628 (52)	10 (40)	
Male	2947 (39)	8 (80)	X^2^ = 7.2044 *p* = 0.0073
Female	4681 (61)	2 (20)		
≤44 years old	2562 (34)	4 (40)	X^2^ = 0.184 *p* = 0.6679
≥45 years old	5066 (66)	6 (60)		
Healthcare Associated			
Total healthcare associated cases	6984 (48)	14 (56)	
Male	3276 (47)	9 (64)	X^2^ = 1.6942 *p* = 0.1930
Female	3708 (53)	5 (36)		
≤44 years old	1074 (15)	4 (29)	X^2^ = 1.8663 *p* = 0.1719
≥45 years old	5910 (85)	10 (71)		
Unknown Exposure Source	**	1 (7)	
Antibiotic Use Within 12 Weeks of CDI Diagnosis			
Yes	4532 (60)	9 (36)	
No		14 (56)	
Unknown	2967 (40) *	2 (8)	
Underlying Conditions			
Cardiovascular disease	1542 (21)	10 (40)	X^2^ = 8.0906 *p* = 0.0883 (note that cardiovascular, cardiopulmonary, malignancy, and transplant were combined owing to small n for UMC)
Diabetes mellitus	1658 (22)	6 (24)	
Cardiopulmonary obstructive disease	1413 (19)	1 (4)	
Renal Disease	1289 (17)	4 (16)	
Neurologic conditions	1223 (16)	4 (16)	
Malignancy	1244 (17)	2 (8)	
Transplant	233 (3)	1 (4)	

* CDC data does not specify no antibiotics taken vs. unknown.

** Not defined by CDC data set.

**Table 2.** UMC sample Ribotypes—Reporting information.

Ribotype	Frequency in UMC Samples	Locations as of 2022	Isolate Sources
002	1	United Kingdom, Romania, Belgium, Germany, Hong Kong, Sweeden, Portugal, Netherlands, Italy, Poland, France	Human, Environment, Livestock,
012	1	Costa Rica, Germany, Switzerland, United Kingdom, United states, Hong Kong	Environment, Human
014	1	Australia, France, Germany, Hungary, Italy, Poland, Romania, Slovakia, South Korea, Spain, United Kingdom, United States, Hong Kong	Enviroment, Human, Livestock
015	1	Bulgaria, France, Germany, Ireland, Netherlands, Romania, United Kingdom, United States Hong Kong	Human
020	1	Belgium, Czechia, France, Germany, Italy, Netherlands, Romania, Spain, United Kingdom, United States, Hong Kong	Environment, Human, Laboratory
027	9	Australia, Belgium, Canada, France, Germany, Hungary, Italy, Japan, Netherlands, Poland, Portugal, Romania, Singapore, South Korea, Spain, United Kingdom, United States, Hong Kong	Companion, Animal, Environment, Food, Human, Laboratory, Livestock
054	1	Germany, United Kingdom, United States	Human, Laboratory
056	1	Germany, United Kingdom, Hong Kong, United States	Human
070	1	Germany, United Kingdom, United States, Hong Kong	Human
076	2	Spain, United States	Human
103	1	France, Germany, United Kingdom, United States, Hong Kong	Human
106	1	France, Germany, Spain, United Kingdom, United States	Human, Laboratory
126	1	France, Germany, Poland, Spain, United Kingdom, United States	Livestock, Human, Environment,
137	1	United Kingdom	Human
160	1	No Information	No information
220	1	German, United Kingdom, Hong Kong	Environment, Human
255	1	United Kingdom, Hong Kong, United States	Human
302	1	No Information	No information
363	1	No Information	No information
813	1	No Information	No information

## Data Availability

Raw data supporting the conclusions of this article will be made available by the authors upon request.

## Abbreviations

The following abbreviations are used in this manuscript:

CDI: Clostridioides difficile infections
UMC: University Medical Center of Southern Nevada
HA-CDI: Healthcare-acquired CDI
CA-CDI: Community-acquired CDI
CDRN: *Clostridioides difficile* Ribotyping Network for England and Northern Ireland
PPIs: Proton pump inhibitors
